# Therapeutic and immunoregulatory effects of water-soluble alkaloids E2-a from *Sophora moorcroftiana* seeds as a novel potential agent against echinococcosis in experimentally protoscolex-infected mice

**DOI:** 10.1186/s13567-018-0596-9

**Published:** 2018-10-04

**Authors:** Yanping Luo, Guochao Zhang, Xun Liu, Miaomiao Yuan, Qi Gao, Haijun Gao, Lixin Ke, Xinxing Zhang, Yanbin Shi, Xingming Ma, Lifeng Zhang, Kaizhong Dong

**Affiliations:** 1 Department of Immunology, School of Basic Medical Sciences, Lanzhou University, Lanzhou, 730000 China; 2 Institute of Pathogen Biology, School of Basic Medical Sciences, Lanzhou University, Lanzhou, 730000 China; 3 Cancer Research Institute, School of Basic Medical Sciences, Southern Medical University, Guangzhou, 510515 China; 4 School of Pharmacy, Lanzhou University, Lanzhou, 730000 China; 5 Key Lab of Preclinical Study for New Drugs of Gansu Province, Lanzhou, 730000 China; 6 Department of Microbiology, Medical College, Northwest University for Nationalities, Lanzhou, 730030 China

## Abstract

Novel compounds and more efficient treatment options are urgently needed for the treatment of cystic echinococcosis (CE), which is caused by *Echinococcus granulosus*. The decoction of *Sophora moorcroftiana* (Fabaceae) has been used to treat parasitosis for years in traditional Tibetan medicine. The aim of this study was to screen insecticidal water-soluble alkaloids from *S. moorcroftiana* seeds and evaluate the therapeutic effects against CE and the immune response induced by the alkaloidal fraction. Low polarity compounds (E2-a) were isolated from water-soluble alkaloid (E2) and matrine and sophocarpine were identified as major components. The E2-a fraction was more effective against protoscoleces than other constituents from *S. moorcroftiana*. After 20 weeks of secondary infection with protoscoleces, mice were orally treated with E2-a (100 mg/kg/day) for 6 weeks to evaluate therapeutic and immunoregulatory activities. Compared with the untreated group, E2-a treatment induced a significant reduction in cyst weight (mean 2.93 g) (*p *< 0.05) and an impaired ultrastructural modification of the cyst. Interestingly, the application of E2-a resulted in a significant increased frequency of CD3^+^CD4^+^ T-cell subsets and decreased frequency of CD3^+^PD-1^+^ T-cell subsets, compared with protoscolece-infected mice without treatment. The E2-a fraction of *S. moorcroftiana* can inhibit the cyst development of CE and boost the specific immune response by reducing the expression of PD-1 and accelerate the cytokine secretion of antigen-specific T-cells. All data suggest the E2-a fraction from *S. moorcroftiana* seeds may be used as a new potential therapeutic option against *E. granulosus* infection.

## Introduction

Cystic echinococcosis (CE) caused by the larval stage of *Echinococcus granulosus* (sensu lato) is a chronic zoonotic parasitic disease which remains a threat to human health. *E. granulosus* has a worldwide distribution, the Mediterranean countries, Russia and China are recorded as being highly endemic [1]. The parasite also infects domestic livestock such as cattle, sheep and horses, leading to serious economic losses in farming and stockbreeding regions [2]. *Echinococcus* has a two-host life cycle, a carnivorous definitive host in which the adult cestode develops in the small intestine and an intermediate host, such as humans and domestic livestock, in which the metacestode develops in the viscera and protoscoleces are produced [3]. The metacestode is a fluid-filled cystic structure that undergoes a sexual multiplication to produce large numbers of protoscoleces [3]. Protoscolices are microscopic larvae that are capable of developing into a sexually mature adult worm in the final host intestine or re-differentiation into hydatids in the intermediate host viscera upon vesicle rupture [3]. Because protoscoleces maintain a longer survivability and are easy to culture in vitro, they were often used to screen the agents against echinococcosis [4]. Recently, four approaches have been recommended to treat CE: observation (watch-and-wait approach) for inactive, clinically silent cysts, chemotherapy with benzimidazoles, percutaneous sterilization and surgery [5]. The preferred treatment option is surgical excision of the parasitic mass for treatment of human CE. To reduce the risk of recurrence, chemotherapy is often employed to complement surgery. Albendazole and mebendazole are generally considered to be the most effective chemotherapeutic drugs against CE and are widely used. However, such chemotherapy does not work well in 20–30% of cases [6]. In addition, adverse side effects have been observed with these compounds [7]. Therefore, novel compounds and more efficient treatment options are necessary. Phytochemicals may be the source of anti-parasitic agents and have received much attention recently [8, 9], suggesting that isolation of active anthelmintic constituents may lead to the discovery of compounds with improved therapeutic efficacy against CE.

*Sophora moorcroftiana* (Fabaceae) is a shrub, which grows at the Yarlung Zangbo River basin in Tibet (China), and is also found in India, Bhutan and Nepalat at altitudes from 3000 to 4500 m, according to the *Flora of China* [10]. The seeds have been used to treat parasitoses for years in traditional Tibetan medicine in China [11]. Previous studies showed that the fat-soluble crude alkaloids from *S. moorcroftiana* seeds in combination with albendazole have protoscolicidal effects [12–14]. However, application of the crude alkaloids alone did not show any inhibition against parasite infection due to its low solubility and an undetermined component with greater toxicity (LD_50_ = 207.8 ± 20.82 mg/kg) [14]. We here isolated the water-soluble alkaloids (Extract 2, E2), and the further fractions, including E2-a and E2-b. Then the in vivo therapeutic effects against CE and cellular immunity induced by the water-soluble alkaloid fraction E2-a were evaluated in an experimental mouse model infected with protoscoleces.

## Materials and methods

### Isolation of alkaloids from *S. moorcroftiana* seeds

*Sophora moorcroftiana* seeds were obtained from Tibet in China and identified by Hongyu Li (School of Pharmacy, Lanzhou University, Lanzhou, China). Alkaloids were extracted as follows [15]: The dried seeds (5 kg) were pulverized, then sieved with a 10-mesh (bore diameter was 1651 μm) steel strainer, soaked in 60% ethanol for 24 h and thereafter extracted at 80 °C for 4 h three times successively. The fluid extract was collected after concentration using a rotary evaporator and was adjusted to a pH of 4 with 12 M HCl. Then the fluid was centrifuged for 10 min at 2000 rpm and the supernatant was collected; pH was adjusted to 12 with 10 M NaOH. After centrifugation for 10 min at 2000 rpm, the yellow precipitate which contained the non-water-soluble alkaloidal fraction (Extract 1, E1) was discarded. Then the supernatant that contained the water-soluble alkaloidal fraction (Extract 2, E2) was extracted with chloroform. The chloroform phase was collected and the solvent was eliminated with a rotary evaporator to obtain dry material (115 g fraction E2). Next, silica gel column chromatography was performed to separate the alkaloid fractions from E2. E2 was dissolved in chloroform for loading. After elution with chloroform and methanol, two fractions were obtained: E2-a (productive rate was 39.76%) with low molecular polarity [main band retention factor (Rf) = 0.93] and E2-b (production rate was 27.83%) with high molecular polarity (main band Rf = 0.54).

### Thin layer chromatography

Analytical thin layer chromatography (TLC) was used for the rapid identification of alkaloid components and carried out on pre-coated TLC plates with silica gel 60 F_254_ (0.2 mm, Merk, Darmstadt, Germany). Briefly, a chromatographic pre-coated silica gel plate was used as the stationary phase. The sample was applied with a sample applicator. The TLC plates were developed in a pre-saturated glass chamber containing a mixture of chloroform/methanol/ammonia-water (25:3:0.8 v/v/v) as the mobile phase. The plate was removed when the edge of the solvent was 7 cm from the original sample position and allowed to blow-dry. After drying, the plates were developed with Dragendorff reagent [16]. Meanwhile, oxymatrine, oxysophocarpine, matrine and sophocarpine were prepared at concentrations of 20 mg/mL in methanol as reference substances. The Rf values for each component of extracts were determined with the following formula: Rf = distance traveled by the solute from the point of application to the center of spot/distance traveled by the solvent front [17].

### High performance liquid chromatography (HPLC)

E2-a was analyzed by HPLC using a Waters HPLC system (Milford, MA, USA) with Waters 1525 binary HPLC pump, Waters 2998 photodiadearry (PDA) detector and VP-ODS-C18 column (5 μm, 250 mm × 4.6 mm) [18]. Isocratic elution was used to analyze all samples in this experiment (flow rate of 1.0 mL/min; UV detection; column temperature 35 °C). The mobile phase was comprised of 25% A (acetonitrile) and 75% B (0.02 mol/L ammonium acetate containing 0.05% triethylamine). As a comparison, a mixture of standards, matrine and sophocarpine were also analyzed with the same HPLC conditions. In the analysis of E2-a, identification of analytes was based on matching the retention times against those of standard compounds and was confirmed by spiking standards to the samples. Matrine and sophocarpine standards were prepared at concentrations of 500 μg/mL in HPLC grade methanol as stock solutions. Work solution contained 50 μg/mL of standard was prepared from the stock standard solution and filtrated by a filter (pore diameter was 0.22 μm). The quantitation analysis was based on the peak area using the internal calibration method. According to the principle, the concentration of a compound is proportional to the peak area provided by HPLC, and therefore the concentration of the compound was determined. The analysis was performed in triplicate.

### Preparation of *E. granulosus* protoscoleces

Isolation and maintenance of *E. granulosus* protoscoleces in vitro were carried out as previously described [9]. In brief, protoscoleces of *E. granulosus* were collected aseptically from liver hydatid cysts of naturally infected sheep slaughtered at the Xining abattoir, Qinghai province, China. Protoscoleces were washed five times in saline and subsequently resuspended with phenol red free Dulbecco’s minimal essential medium (DMEM), containing 2 mM l-glutamine, 25 mM HEPES, 100 U/mL penicillin and 100 μg/mL streptomycin, supplemented with 10% fetal calf serum. The survival rate of the protoscoleces exceeded 95% after these procedures by the trypan blue exclusion test.

### In vitro drug treatment of *E. granulosus* protoscoleces

One milliliter of DMEM medium containing 100 protoscoleces was transferred to a 24-well plate and cultured together with the following fractions or drugs: E2-a, E2-b, E2, matrine and sophocarpine, respectively. The final concentration of each drug was 0.5 mg/mL. Protoscoleces incubated with or without albendazole (0.01 mg/mL) were used as controls. The plates were incubated at 37 °C under a 5% CO_2_ atmosphere for 7 days. Protoscoleces were observed each day in triplicates with an optical microscope (Olympus IX71, Tokyo, Japan) [9]. Additionally the trypan blue exclusion test was used to evaluate the viability of protoscoleces. After treatment, the viability of protoscoleces was calculated. Using the same method, the protoscolicidal effects of E2-a with the final concentration of 8 mg/mL to 0.0125 mg/mL (1:2 dilution steps) were assessed. To reduce the bias as much as possible, protoscolex viability was observed by two experimenters under double-blind conditions. Each experiment was repeated twice.

### Effects of E2-a on *E. granulosus* protoscoleces in experimentally infected mice

Specific pathogen free (SPF) female NIH mice were purchased from Lanzhou Institute of Biological Products Co., Ltd (Lanzhou, China) and body weight was 20 ± 2 g at the start of the experiments. Mice were maintained in the SPF Grade Trial Animal Center (Lanzhou University, Lanzhou, China). Mice received free access to food and water throughout the study. All experiments were carried out according to the protocols (2015-03-002) approved by the Institutional Animal Care and Use Committee of Lanzhou University.

Five mice without protoscolex infection were intragastrically administrated with PBS (0.4 mL/mouse) as a non-infected group. Fifteen mice were respectively infected with an intraperitoneal injection of 5000 protoscoleces in 0.7 mL of DMEM medium. At 20 weeks post-infection, those mice were randomly divided into three groups (five mice/group). Mice were daily intragastrically given PBS (0.4 mL/mouse) as an untreated group, while mice were daily administrated with albendazole (100 mg/kg) or E2-a (100 mg/kg) as treatment groups of albendazole and E2-a, respectively. After 6 weeks, all mice were euthanized by cervical dislocation under narcosis with 0.3% pentobarbital sodium (10 mL/kg) by intraperitoneal injection. The parasite tissues were carefully removed from the peritoneal cavity by necropsy and weighed by electronic balance. The hydatid cysts from each group were collected randomly in 10% formalin and processed for histopathology. Sections were prepared and stained with haematoxylin and eosin (H&E). Then pathologic changes in the cyst wall were examined by light microscopy.

### Scanning electron microscopy (SEM)

The hydatid cysts from *E. granulosus* protoscolece-infected mice with or without treatment were collected randomly and were processed for SEM [19]. Briefly, the cysts were fixed with 2.5% glutaraldehyde for more than 30 min at 4 °C. After washing away the primary fixative with PBS, the tissues were fixed again with 1% osmic acid away from light for 30 min at 4 °C. Afterwards, the fixative was discarded. The samples were then dehydrated in various concentrations of alcohol (50, 70, 80, 90%, and 100%) for 10 min at 4 °C. Subsequently, they were immersed in 2% isoamyl acetate for 20 min. Finally, they were sputter-coated with gold after drying by critical point drying method and viewed on a JEOL JSM-5600LV scanning electron microscope.

### Flow cytometry (FCM)

Spleens from *E. granulosus* protoscolex-infected mice with or without treatment were aseptically removed and forced through a 200-mesh nylon strainer. Then, single-cell suspensions were prepared with lymphocyte-M (Dakewe Biotech Company Limited, Shenzhen, China) density gradient centrifugation and flow cytometry (FCM) was performed as described previously [20]. After washing with flow cytometry staining buffer, cell staining was performed according to standard protocols. The cells were stained with antibody cocktail consisting of PerCP-eFluor710-conjugated anti-CD3 (17A2), PE-conjugated anti-CD4 (GK1.5), APC-conjugated anti-CD8 (53–6.7) and FITC-conjugated anti-PD-1 (J43) for 30 min at 4 °C under darkness. Then the cells were washed with flow cytometry staining buffer twice. All of the antibodies for FCM staining were purchased from eBioscience (San Diego, CA, USA). Flow cytometry was performed by ACEA NovoCyte (ACEA Biosciences, Inc., Hangzhou, China) and the results were analyzed with NovoExpress.

### Antibody array

The splenocytes from mouse (5 × 10^6^ cells/well) were seeded into 24-well tissue culture plates and stimulated with or without 50 μg/mL of whole hydatid fluid antigen from hydatid cysts of a mouse. After incubation at 37 °C in 5% CO_2_ for 72 h, the supernatants were collected and stored at −80 °C. A QAM-CYT-1 antibody array (RayBiotech, Norcross, GA, USA) was used to detect 20 cytokines.

### Liver and kidney toxicity of E2-a in vivo

Six uninfected female BALB/c mice were purchased from the Laboratory Animal Center of Lanzhou University and used to investigate the toxicity of E2-a. The mice were maintained at the same conditions with NIH mice. Three mice received intragastric administration of PBS (0.4 mL/mouse) and the other three mice received intragastric administration of E2-a (100 mg/kg) daily for 6 weeks. Then blood samples were taken before euthanasia by cervical dislocation under narcosis. The serum was collected and biochemical indexes such as total bilirubin, direct bilirubin, indirect bilirubin, total protein, albumin, globulin, alkaline phosphatase, alanine aminotransferase, aspartate amino transferase, urea and creatinine were detected. Afterwards, all livers and kidneys were collected and soaked in 10% formalin and processed for histopathology.

### Statistical analysis

Statistical analysis was done with SPSS 11.5 software. One-way analysis of variance (ANOVA) was used to analyze the data on the effects of E2-a against *E. granulosus* and nonparametric test of the Mann–Whitney U test was used for serum biochemical findings in BALB/c mice. Data are shown as mean ± standard deviation of the mean (SD). *p*-value < 0.05 was considered statistically significant.

## Results

### TLC and HPLC examination of alkaloids

**Figure 1:**
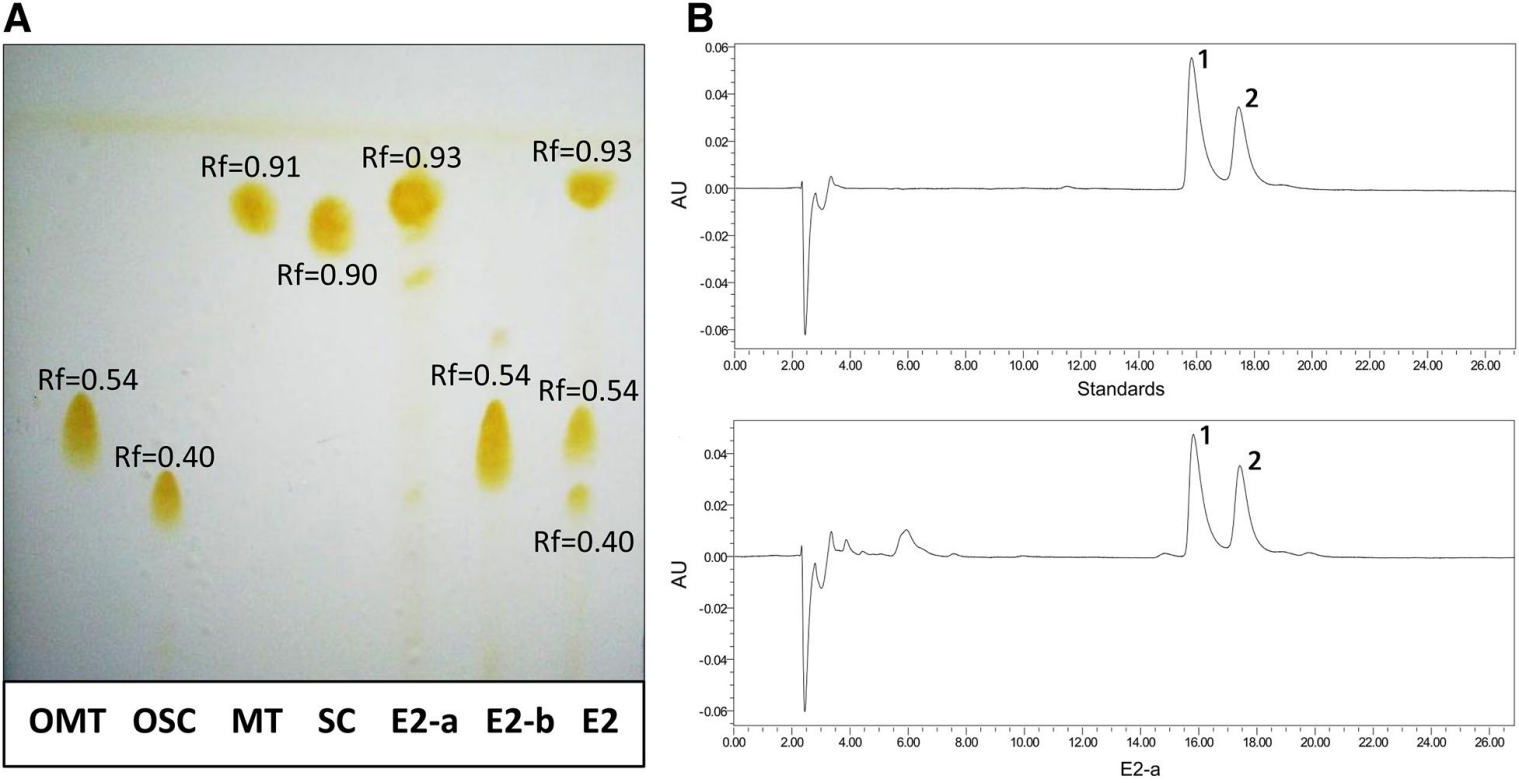
**The constituent of** ***S. moorcroftiana*** **seed alkaloids were analyzed with thin layer chromatography (TLC) and high performance liquid chromatography (HPLC). A** The TLC shows E2-a with Rf 0.93 and E2-b with Rf 0.54 in comparison with the lanes of oxymatrine (OMT), oxysophocarpine (OSC), matrine (MT) and sophocarpine (SC), respectively. **B** The typical HPLC chromatograms of a mixture of standards with matrine and sophocarpine (upper panel) and E2-a (lower panel) are shown. The peaks of 1 and 2 are matrine and sophocarpine, respectively.

To identify the bioactive chemical compounds of the alkaloids from *S. moorcroftiana* seeds, TLC was performed as a qualitative method to document the extract constituents. The TLC results revealed three bands with Rf value of 0.93, 0.54 and 0.40 respectively (Figure 1). The previous study showed that oxymatrine (OMT), oxysophocarpine (OSC), matrine (MT) and sophocarpine (SC) were identified in *S. moorcroftiana* seeds [18], and therefore these substances were used as reference compounds. The results of TLC show Rf values of approximately 0.9 for matrine and sophocarpine, and Rf values of approximately 0.5 for oxymatrine. Thus, the main band of E2-a with Rf 0.93 and the main band of E2-b with Rf 0.54 were obtained from E2 by column chromatography suggesting that E2-a could contain matrine and sophocarpine and E2-b could contain oxymatrine and oxysophocarpine.

To identify the main constituent of E2-a, HPLC was carried out (Figure 1B). The results indicate that matrine and sophocarpine are major components of E2-a. The calculated ratios of matrine and sophocarpine were 43% and 26%, respectively.

### E2-a showed strong protoscolicidal effects in vitro

**Figure 2:**
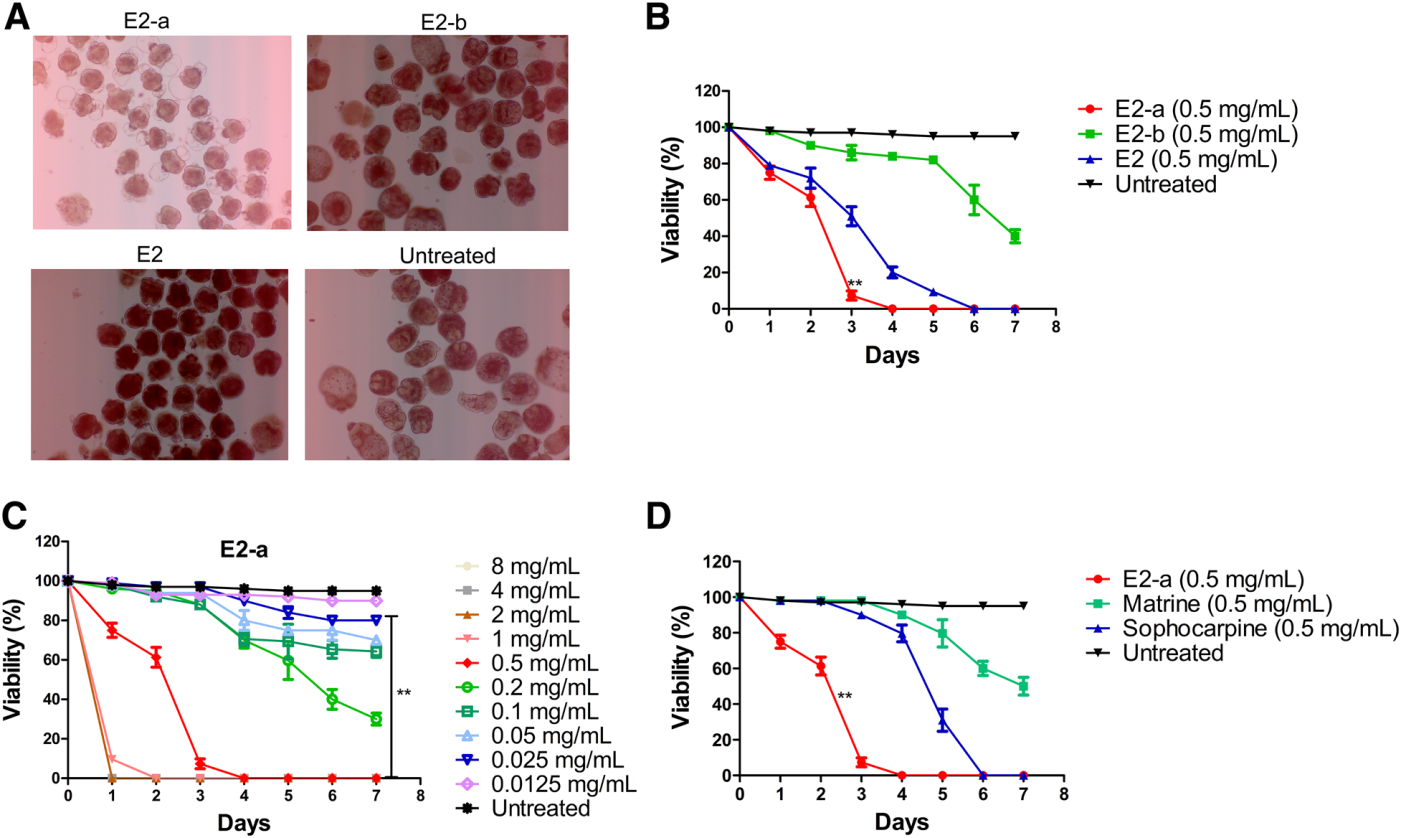
**Viability of protoscoleces in vitro was assessed with trypan blue staining.** After treatment with alkaloidal fractions (0.5 mg/mL) from *S. moorcroftiana* seeds for 3 days, the representative morphology of protoscoleces are presented under light microscopy (**A**). After treatment with alkaloidal fractions (0.5 mg/mL) from *S. moorcroftiana* seeds, the viability of protoscoleces are shown (**B**). **p < 0.01 for E2-a vs other groups. After treatment with E2-a, the viability of protoscoleces are shown (**C**). **p < 0.01 for E2-a (concentration ≥ 0.025 mg/mL) vs untreated. After treatment with E2-a (0.5 mg/mL), matrine (0.5 mg/mL) and sophocarpine (0.5 mg/mL), the viability of protoscoleces are shown respectively (**D**). **p < 0.01 for E2-a vs other groups.

To test active constituents from *S. moorcroftiana* seeds against *E. granulosus*, the protoscolicidal effects of E2-a, E2-b and E2 were assessed in vitro. The results show that E2-a possessed higher protoscolicidal effects than the other extracts. Figure 2A shows the morphological changes of protoscoleces at day 3 after treatment with the extracts (0.5 mg/mL) and trypan blue staining. Protoscoleces in the medium without extracts shows normal morphology and vitality during the culture period. Most protoscoleces treated with E2-a were shrunken, with vacuolization in the scolex and soma regions. However, E2-b or E2 just led to a low slight shrinking of protoscoleces without vacuolization (Figure 2A). The results of viability tests for 7 days reveal that E2-a induced lower viability compared with the untreated protoscoleces at day 3 (*p* < 0.01) and all protoscoleces died at day 4 and 6 after treatment with E2-a (0.5 mg/mL) and E2 (0.5 mg/mL), respectively. Nevertheless, 40% of the protoscoleces survived after treatment with E2-b (0.5 mg/mL) until day 7 (Figure 2B). This data shows that E2-a induces stronger protoscolicidal effects than the other extracts.

Further, the anti-echinococcal activity of different concentrations of E2-a was investigated. All protoscoleces were killed at 24 h after exposure to E2-a (≥ 2 mg/mL). Treatment with E2-a at 1 mg/mL for 3 days and 0.5 mg/mL for 4 days led to 100% mortality of the parasites. E2-a (concentration ≥ 0.025 mg/mL) induced decreased viability of protoscoleces compared with the untreated protoscoleces at day 7 (*p* < 0.01) (Figure 2C).

To compare protoscolicidal effects of E2-a and compounds which exist in the E2-a fraction, the protoscoleces were incubated with E2-a, matrine and sophocarpine (all at 0.5 mg/mL). The protoscolicidal efficacy of E2-a was superior to sophocarpine or matrine (*p* < 0.01) (Figure 2D).

### Therapeutical effects of E2-a on *E. granulosus* metacestodes in vivo

**Figure 3:**
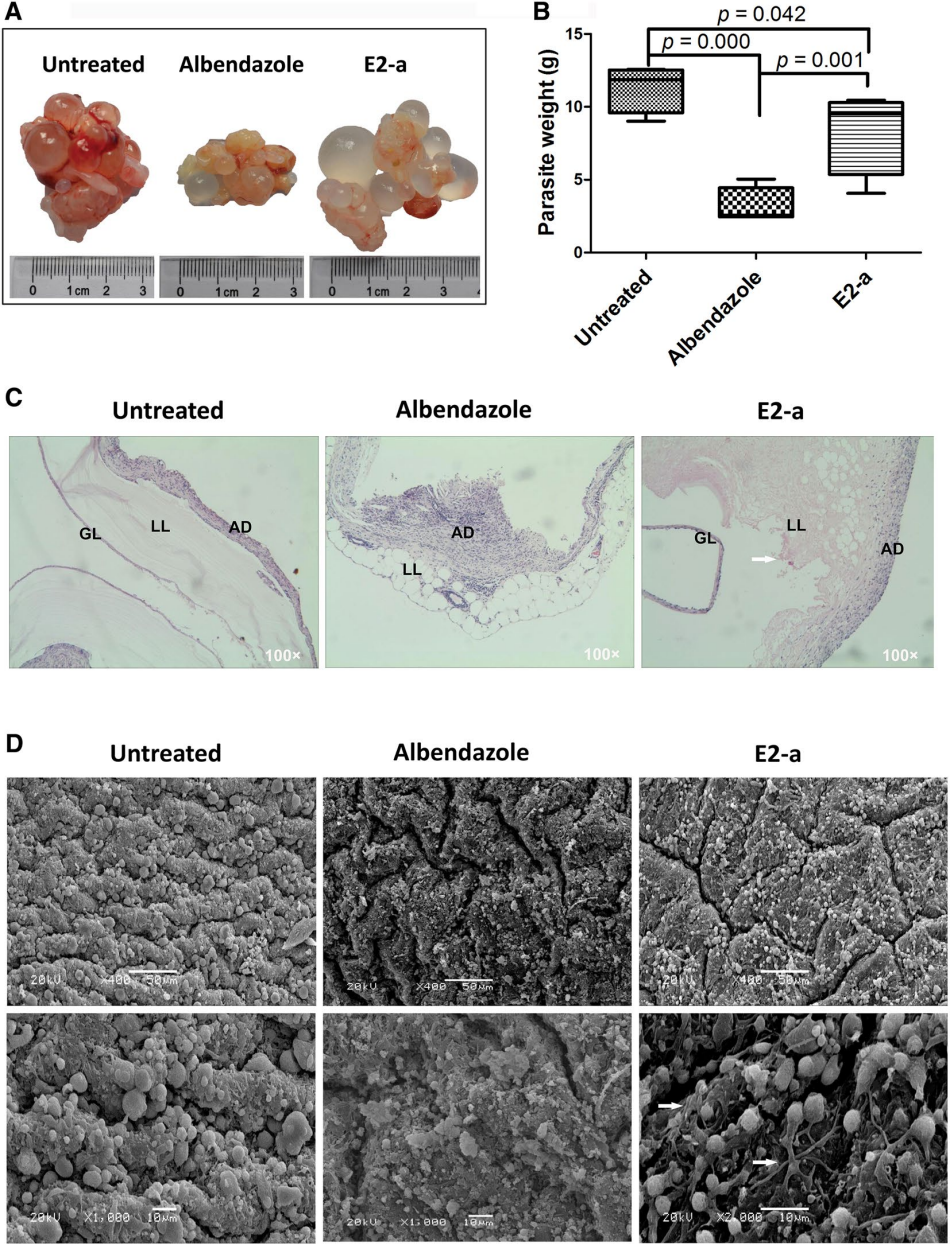
**The E2-a shown therapeutical effects against** ***Echinococcus granulosus*** **sensu stricto in vivo.** Mice (*n* = 5) were intraperitoneally injected with protoscoleces. At 20 weeks after infection, the treated mice were intragastrically administrated with E2-a (100 mg/kg) and albendazole (100 mg/kg) respectively, untreated mice received PBS alone each day for 6 weeks. The representative hydatid cyst (**A**), the average weight of all cysts per group (*n* = 5) (**B**), the representative pathological characteristics (H&E, 10 × 10) (**C**) and the ultrastructure (SEM) **D** of the metacestodes are shown respectively. The bar of the upper panel was 50 μm and the bar of the under panel was 10 μm. GC: germinal layer; LL: laminated layer; AD: adventitia.

Unilocular fluid-filled cysts were found in each experimental mouse and representative hydatid cysts are shown in Figure 3A. Rich blood vessels were observed at the surface of cysts and increased adhesion among cysts in the untreated group in contrast to the E2-a treated group. The treatment with E2-a led to a significantly lower weight of cysts (8.40 ± 2.93 g) compared with the untreated group (11.33 ± 1.64 g) (mean reduction of 2.93 g, anti-hydatid rate of 74%) (*p* < 0.05), although the efficacy was lower when compared to the albendazole-treated group (*p *< 0.01) (Figure 3B).

The microscopic structures of hydatid cyst with H&E staining are shown in Figure 3C. A consecutive germinal layer, texture clear laminated layer and clear boundary between laminated layer and adventitia were observed in the untreated group. In the E2-a treated group, the hydatid cyst lost the characteristic cellular grainy texture and exhibited a thick but loosened and vacuolated laminated layer. Although the germinal membrane was still intact, it partly detached from the laminated layer in E2-a-treated metacestodes. Besides, the view showed the degeneration of the adventitia fiber and the indistinct boundary between laminated layer and pericystic adventitia in the E2-a group. SEM showed that typical structure of *E. granulosus* cyst appeared in the untreated mice, whereas the drug-treated mice lost this typical structure. In the germinal layer, increased fiber and deep fractures were found in the E2-a-treated group, indicating that the germinal layer was impaired (Figure 3D).

### E2-a increased the frequency of CD3^+^CD4^+^ T cells in protoscolex-infected mice

**Figure 4:**
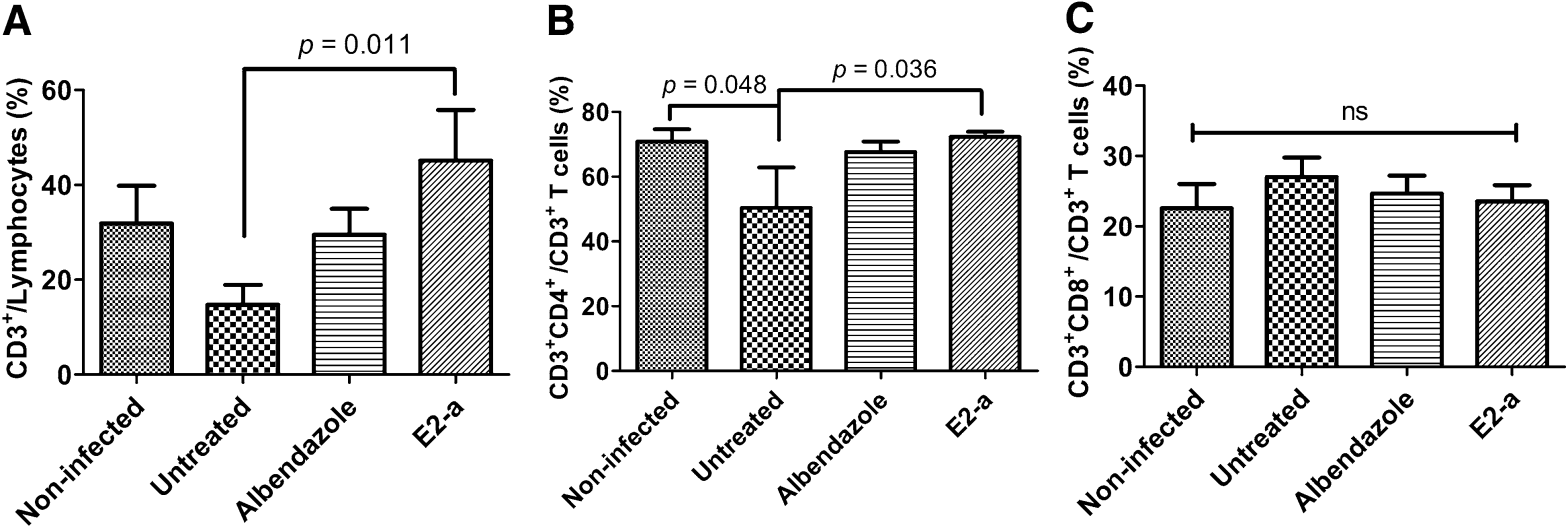
**T-cell subpopulations were analyzed by flow cytometry (FCM).** Splenocytes were stained with PerCP-eFluor710-conjugated anti-CD3, PE-conjugated anti-CD4 and APC-conjugated anti-CD8. CD3^+^ T cells, CD3^+^CD4^+^ and CD3^+^CD8^+^ T cells then were detected via FCM. The ratio of CD3^+^ T cells in lymphocytes (**A**), the ratio of CD3^+^CD4^+^ T cells in CD3^+^ T cells (**B**), and the ratio of CD3^+^CD8^+^ T cells in CD3^+^ T cells (**C**) are shown respectively. Data collected from five mice per group were expressed as mean ± SD.

As cellular immune response is important against CE, [21], the T-cell subpopulation induced by E2-a was analyzed in this study. The data of FCM show that the ratio of CD3^+^CD4^+^ T-cell subpopulation in untreated mice was lower than that in the non-infected group (*p *= 0.048). The further analysis showed that E2-a induced a significant increase in percentage of total CD3^+^ T-cell (*p *= 0.011) and CD3^+^CD4^+^ T-cell subpopulations compared with the untreated group (Figures 4A and B). However, No statistical difference of CD3^+^CD8^+^ T-cell subpopulations was found between each group (*p* > 0.05) (Figure 4C).

### E2-a reduced the frequency of PD-1^+^ T cells in protoscolex-infected mice

**Figure 5:**
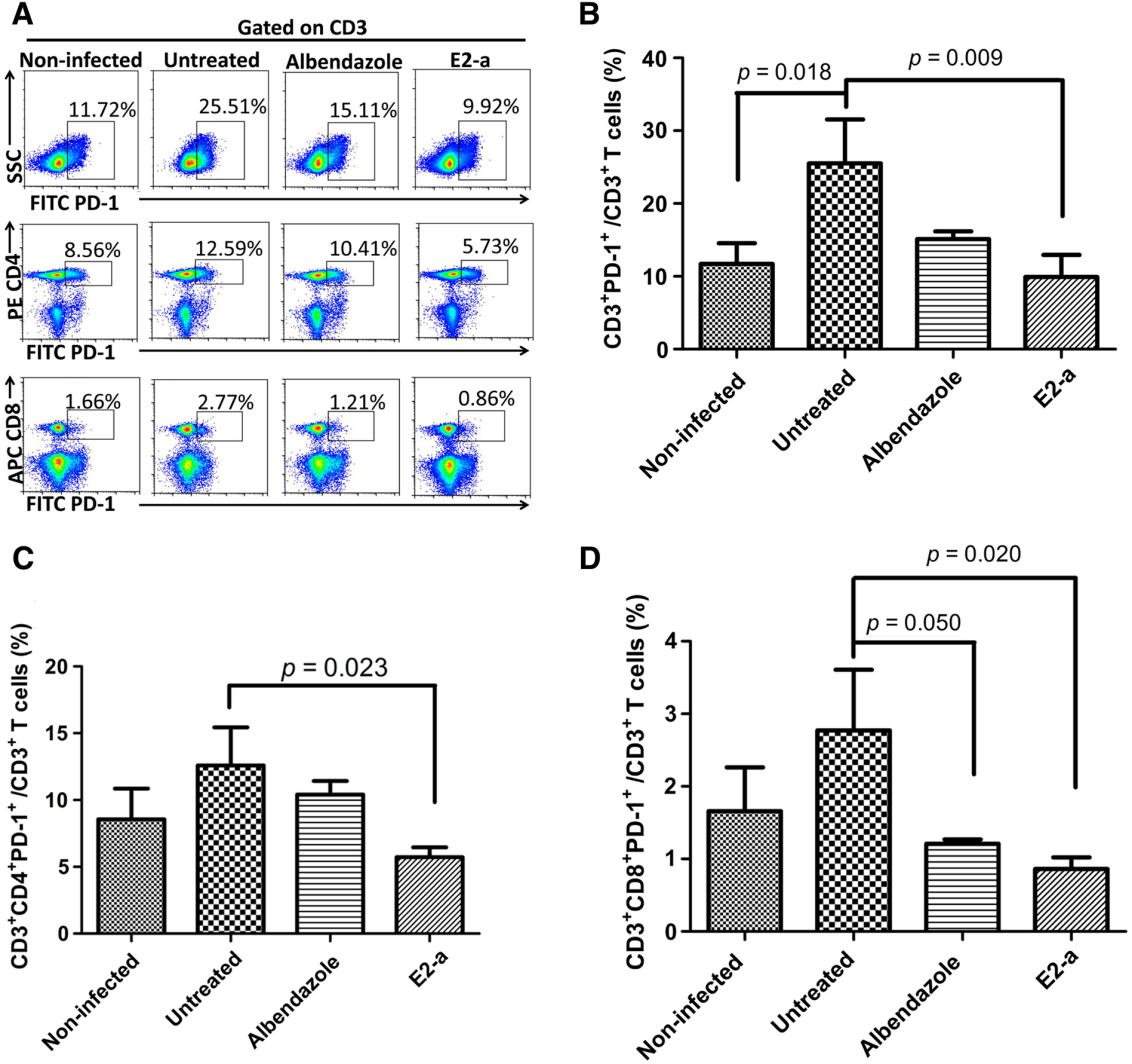
**PD-1**^**+**^ **T cells were counted with FCM.** Splenocytes from experimental mice were stained with PerCP-eFluor710-conjugated anti-CD3, PE-conjugated anti-CD4, APC-conjugated anti-CD8 and FITC-conjugated anti-PD-1. CD3^+^PD-1^+^ T cells, CD3^+^CD4^+^PD-1^+^ and CD3^+^CD8^+^PD-1^+^ T cells were detected via FCM. The representative results were depicted (**A**). The ratio of CD3^+^PD-1^+^ T cells in CD3^+^ T cells (**B**), the ratio of CD3^+^CD4^+^PD-1^+^ T cells in CD3^+^ T cells (**C**) and the ratio of CD3^+^CD8^+^PD-1^+^ T cells in CD3^+^ T cells (**D**) are shown respectively. Data collected from five mice per group were expressed as mean ± SD.

As an inhibitory receptor, PD-1 is overexpressed in numerous chronic infectious diseases, including hydatid disease and tumors, and it has been shown to contribute to maintaining peripheral tolerance and immune evasion [22–25]. Compared with non-infected mice, the frequency of CD3^+^PD-1^+^ T-cell subpopulation distinctly increased in protoscolex-infected mice without treatment (*p *= 0.018) (Figure 5B). However, mice treated with E2-a for 6 weeks showed a significant reduction in frequency of the CD3^+^PD-1^+^ T-cell subpopulation compared with the untreated group (*p *= 0.009). The analysis of CD4 and CD8 molecule-expression revealed that E2-a induced a decline in PD-1^+^ T cells depending on the decrease of both CD4^+^PD-1^+^ (*p *= 0.023) (Figure 5C) and CD8^+^PD-1^+^ T cells (*p *= 0.020) (Figure 5D).

### Cytokine expression

**Figure 6:**
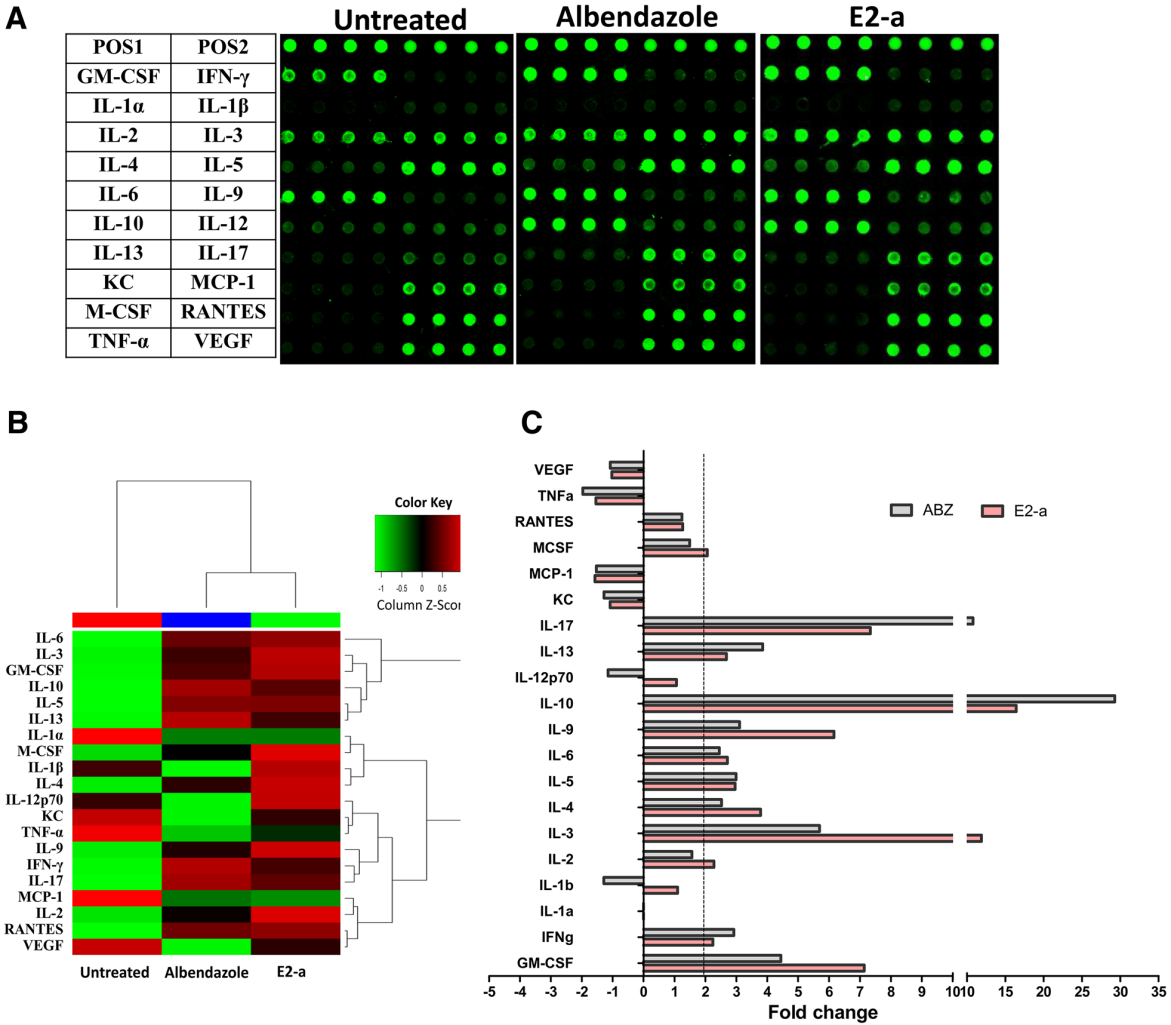
**Cytokine expression in culture suparnatant of splenocytes was measured by antibody array.** The array images (**A**) and the heat map of cytokine expression (**B**) are shown respectively. **C** The fold change of cytokine present more than twofold increased expression in treatment groups.

To analyze immune effects induced by E2-a, an antibody array was used to examine cytokine levels (Figure 6A). The differences of cytokines in each group could be observed visually on a heat map (Figure 6B). Compared with the untreated control, E2-a induced an increased expression more than twofold in Th1-Type (IFN-γ, IL-2), Th2-Type (IL-4, IL-5, IL-6, IL-10, IL-13), Th17-Type (IL-17), Th9-Type (IL-9) and other cytokines (IL-3, GM-CSF, M-CSF). Meanwhile, ABZ could also induce an increased expression more than twofolds in Th1-Type (IFN-γ), Th2-Type (IL-4, IL-5, IL-6, IL-10, IL-13), Th17-Type (IL-17), Th9-Type (IL-9) and other cytokines (GM-CSF). However, the levels of IL-2, IL-4, IL-9, IL-3, GM-CSF and M-CSF in the E2-a group were higher than that in the ABZ group, and the levels of IFN-γ, IL-10, IL-13 and IL-17 in the ABZ group were higher than that in the E2-a group (Figure 6C).

### Liver and kidney toxicity of E2-a in vivo

**Figure 7:**
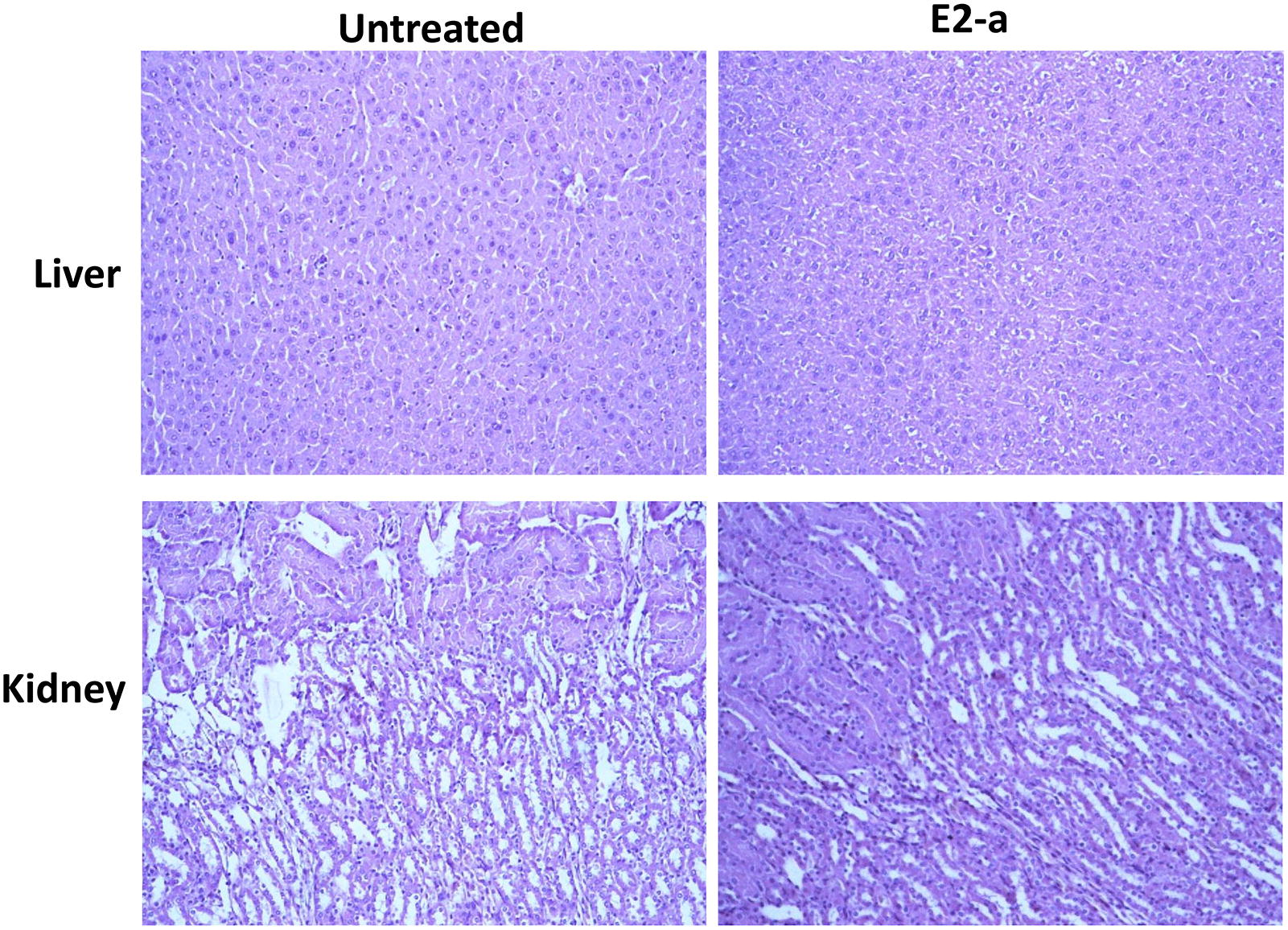
**Histopathology of liver and kidney from mice treated with E2-a was observed under light microscopy.** The BALB/c mice were intragastrically given PBS (0.4 mL/mouse) or E2-a (100 mg/kg) every day for 6 weeks. Afterwards, all livers and kidneys were sectioned and stained with H&E after fixation in 10% formalin. The representative images were shown. The top shows livers and the lower panel shows kidneys.

To investigate in vivo side effects of the extract on livers and kidneys, inbred strain BALB/c mice (three mice per group) were used. The pathological examination was performed in livers and kidneys of mice that had received E2-a treatment for 6 weeks. The microscopy shows that no distinct pathological changes were observed in E2-a-treated mice (Figure 7). Although serum creatinine increased, direct bilirubin slightly decreased in the mice of the E2-a treated group, there was no statistical difference compared with the mice of the untreated group (*p* > 0.05). However, serum alkaline phosphatase and urea in the mice treated with E2-a decreased compared with the untreated mice (*p* = 0.05) (Table 1). Besides, none of the mice presented any abnormal manifestation or death during the experiment.

**Table 1:** **Serum biochemical findings in BALB/c mice treated with E2-a for 6** **weeks (*n*** **=** **3)**

BALB/c mice	Untreated	E2-a
Total bilirubin (μM/L)	1.133 ± 1.206	0.667 ± 0.651
Direct bilirubin (μM/L)	0.467 ± 0.416	0.200 ± 0.200
Indirect bilirubin (μM/L)	0.667 ± 0.987	0.633 ± 0.651
Total protein (g/L)	55.373 ± 3.622	53.420 ± 3.206
Albumin (g/L)	33.733 ± 1.124	32.467 ± 0.907
Globulin (g/L)	21.633 ± 2.610	21.033 ± 2.303
Alkaline phosphatase (U/L)	208.333 ± 4.041	151.000 ± 9.000^*^
Alanine aminotransferase (U/L)	33.333 ± 7.638	38.333 ± 4.726
Aspartate amino transferase (U/L)	139.667 ± 30.172	150.000 ± 35.763
Urea (mM/L)	9.340 ± 1.287	7.667 ± 0.040^*^
Creatinine (μM/L)	11.000 ± 7.211	18.000 ± 1.000

* *p* = 0.01 for E2-a vs untreated.

## Discussion

CE is a disease caused by *E. granulosus* and it still needs improved chemotherapy regimens. In our previous work, we showed that total fat-soluble alkaloids isolated from *S. moorcroftiana* exhibited protoscolicidal effects and the combination of alkaloids and albendazole had significant additive effects [14]. However, treatment with crude alkaloids alone did not show inhibition against parasite infection due to its low solubility and undetermined drug components [14]. In this study, we isolated the water-soluble alkaloid E2 fraction from *S. moorcroftiana,* and then the E2-a with low polarity and the E2-b with high polarity were obtained from the E2 fraction. In in vitro biological assays, the E2-a displayed higher protoscolicidal activity on protoscoleces than that of E2-b and E2. Plentiful shrunken protoscoleces with vacuolation were observed in the protoscoleces treated with E2-a. Furthermore, in vivo E2-a exhibited significant therapeutical effects in experimentally infected mice with *E. granulosus* protoscoleces. In addition, E2-a led to a decreased weight of cysts and morphological alterations in both germinal layer and laminated layer.

In the current study, we isolated alkaloid E2-a fraction and found it was an effective alkaloidal fraction against protoscoleces. The main effective ingredients (69%) of E2-a were matrine and sophocarpine, which presented stronger protoscolicidal activity in vivo and obvious inhibiting effects against growth of *E. granulosus* cyst in experimentally infected mice. Matrine, a main active alkaloid of *Sophora flavescens*, was found to inhibit *Cryptosporidium* infection [26] and had obvious effects against the growth of *E. multilocularis* cyst in experimentally infected mice [27, 28]. Sophocarpine also showed significant anti-parasite effects for nematodes in mice [29]. However, in the present study, the extract and compound concentrations tested in vitro were extremely high. The reason might be that E2-a was a crude mixture isolated from *S. moorcroftiana* seeds. The E2-a extract may contain some compounds with anthelmintic activity with low content. In any case, E2-a was the more efficient fraction of *S. moorcroftiana* seeds, and its components matrine and sophocaprine were slightly active. However, oxymatrine and oxysophocarpine, the main ingredients of E2-b, showed no anti-parasite effects. It was suggested that the oxygen group in the alkaloid molecule might affect the activity of compounds [26–29].

The distinguishing feature of the host-parasite interaction is that chronic infection co-exists with a detectable immune response against the parasite [30]. The cellular immune response is thought to protect against *E. granulosus* infection [31]. However, CE could cause changes in T-cell subpopulations. It has been reported that in protoscolex-infected mice, there were higher percentages of CD3^+^ and CD4^+^ cells in peripheral blood, and CD8^+^ cells in the spleen compared with non-infected mice [32]. Symptomatic hydatid patients had proportionally fewer CD3^+^CD8^+^ lymphocytes in peripheral blood than the healthy controls [33]. In cattle with progressive hydatid cysts, CD8^+^ cells were predominant in the pericystic adventitia and a relatively small number of CD4^+^ cells in the same area [34]. The alterations of T cell ratios were not instrumental in the immune response against CE, but might be modulated through therapies. In this study, protoscolex-infected mice with E2-a treatment showed a significant increasing frequency of CD3^+^CD4^+^ T cells, suggesting that the role of E2-a against CE may be correlated with boosted CD4^+^ T-cell subpopulation responses.

The inhibitory receptor programmed death 1 (PD-1), also known as CD279 has aroused general concern because regulating the balance between T cell activation, tolerance, and immunopathology [35]. PD-1 expressed on the surface of T cells was identified as a marker for T cell exhaustion [36]. As a receptor, PD-1 bound its ligands PD-L1 or PD-L2 to play important roles in regulating immune defenses against pathogens especially during chronic infection. Barber et al. reported that PD-1 selectively up-regulates by the exhausted T cells in mice chronically infected with LCMV and blockage of the PD-1/PD-L1 inhibitory pathway could enhance T-cell responses [22]. Subsequently, a number of data show PD-1 leads to exhausted T cells in diverse chronic infections, such as HBV [37, 38], HIV [39, 40], *Mycobacterium tuberculosis* [41, 42]. In parasite-infected disease, PD-1 has been primarily investigated in malaria. Butler et al. reported that infection of humans with *Plasmodium falciparum* resulted in higher expression of PD-1 associated with T cell dysfunction and in vivo blockade of PD-L1 enhanced protective immune responses in mice [43]. Joshua et al. used an experimental malaria model to show that PD-1 mediates distinct reduction in numbers and function of parasite-specific CD8^+^ T cells [44]. In addition, T cell exhaustion was also observed in other parasite infections like leishmaniasis [45] and toxoplasmosis [46]. The exhausted PD-1^+^ T cells were also observed in the alveolar echinococcosis microenvironment and restoring the cells was approved as a strategy for cancer treatment [25, 47]. In this experiment, we found the highest percentage of CD3^+^PD-1^+^ T cells in protoscolex-infected mice without treatment. This suggests the existence of T cell exhaustion in the echinococcosis microenvironment which is involved in the pathogenesis of this disease and associated with elevated CE. Reducing PD-1^+^ T cells may represent a major breakthrough for the treatment of CE. Furthermore, E2-a treatment reduced a low frequency of PD-1^+^ T cells and low cyst weight, suggesting it could be possible that E2-a functions by reducing PD-1^+^ T cells. It has been found that oxymatrine down-regulates peripheral blood HBV-specific CTL surface PD-1 expression in patients with chronic hepatitis [48]. However, there was no data reported that sophocarpine had an influence on the expression of PD-1. Thus, we speculate that the key ingredient from E2-a reversing T cell exhaustion induced by *E. granulosus* is likely matrine, the metabolite of oxymatrine, in combination with sophocarpine.

As a receptor with inhibitory effect, increased PD-1 expression could down-regulate immunity in which cytokines were essential participators [43]. In the present study, high-level expressions of IFN-γ, IL-4, IL-5, IL-6, IL-9, IL-10, IL-13, IL-17 and GM-CSF were detected in ABZ-treated mice and E2-a-treated mice. The most striking finding was that E2-a induced a low expression of PD-1 and an increased expression of hydatid fluid antigen-specific Th1-Type (IFN-γ, IL-2), Th2-Type (IL-4, IL-5, IL-6, IL-10, IL-13), Th17-Type (IL-17), Th9-Type (IL-9) and other cytokines (IL-3, GM-CSF, M-CSF), similar to those found in the ABZ-treated mice. Especially, the expression levels of GM-CSF, IL-3, IL-9, IL-10 and IL-17 increased more than five fold in the E2-a-treated mice. The down-expression of PD-1 that induced by E2-a and ABZ generated an active role to relieve immunosuppression, which could contribute to the proliferation of lymphocytes and production of cytokines by lymphocytes. The cytokine milieu of the spleen lymphocytes showed a Th1, Th2 and Th17 balance, rather than the bias towards one of the Th phenotypes. More precisely, the Th1 and Th17 type of immune response may be responsible for the increased restriction of parasite growth, and Th2 type of immune response may be conducive to antibody production against CE. The E2-a fraction from *S. moorcroftiana* seeds, as a new agent, may elevate Th-cell immune response by inducing cytokine secretion for the control of CE.

In conclusion, the low polarity compound E2-a isolated from *S. moorcroftiana* seeds exhibited a definite scolicidal activity, and boosted the specific immune response by reducing the expression of PD-1, up-regulating the ratio of CD4^+^ T-cell subpopulation and accelerating the cytokine secretion of antigen-specific T-cells. Therefore, the E2-a fraction may be used as a new potential therapeutic option against *E. granulosus* infection.

## Abbreviations

SPF: specific pathogen free
CE: cystic echinococcosis
*E. granulosus*: *Echinococcus granulosus*
TLC: analytical thin layer chromatography
Rf: retention factor
HPLC: high performance liquid chromatography
DMEM: Dulbecco’s minimal essential medium
H&E: haematoxylin and eosin
SEM: scanning electron microscopy
FCM: flow cytometry
ANOVA: one-way analysis of variance
SD: standard deviation
OMT: oxymatrine
OSC: oxysophocarpine
MT: matrine
SC: sophocarpine
PD-1: programmed death 1
